# FDG-PET/CT Imaging Predicts Histopathologic Treatment Responses after Neoadjuvant Therapy in Adult Primary Bone Sarcomas

**DOI:** 10.1155/2010/143540

**Published:** 2010-04-18

**Authors:** Matthias R. Benz, Johannes Czernin, William D. Tap, Jeffrey J. Eckardt, Leanne L. Seeger, Martin S. Allen-Auerbach, Sarah M. Dry, Michael E. Phelps, Wolfgang A. Weber, Fritz C. Eilber

**Affiliations:** ^1^Ahmanson Biological Imaging Division, Department of Molecular and Medical Pharmacology, David Geffen School of Medicine, University of California Los Angeles, Los Angeles, CA 90095-1782, USA; ^2^Division of Medical Oncology, David Geffen School of Medicine, University of California Los Angeles, Los Angeles, CA 90095-1782, USA; ^3^Department of Orthopedic Oncology, David Geffen School of Medicine, University of California Los Angeles, Los Angeles, CA 90095-1782, USA; ^4^Department of Radiology, David Geffen School of Medicine, University of California Los Angeles, Los Angeles, CA 90095-1782, USA; ^5^Department of Pathology, David Geffen School of Medicine, University of California Los Angeles, Los Angeles, CA 90095-1782, USA; ^6^Abteilung Nuklearmedizin, University of Freiburg, 79106 Freiburg, Germany; ^7^Division of Surgical Oncology, David Geffen School of Medicine, University of California Los Angeles, Los Angeles, CA 90095-1782, USA

## Abstract

*Purpose*. The aim of this study was to prospectively evaluate whether FDG-PET allows an accurate assessment of histopathologic response to neoadjuvant treatment in adult patients with primary bone sarcomas. *Methods*. Twelve consecutive patients with resectable, primary high grade bone sarcomas were enrolled prospectively. FDG-PET/CT imaging was performed prior to the initiation and after completion of neoadjuvant treatment. Imaging findings were correlated with histopathologic response. *Results*. Histopathologic responders showed significantly more pronounced decreases in tumor FDG-SUVmax from baseline to late follow up than non-responders (64 ± 19% versus 29 ± 30 %, resp.; *P* = .03). Using a 60% decrease in tumor FDG-uptake as a threshold for metabolic response correctly classified 3 of 4 histopathologic responders and 7 of 8 histopathologic non-responders as metabolic responders and non-responders, respectively (sensitivity, 75%; specificity, 88%). *Conclusion*. These results suggest that changes in FDG-SUVmax at the end of neoadjuvant treatment can identify histopathologic responders and non-responders in adult primary bone sarcoma patients.

## 1. Introduction

Positron emission tomography (PET) with the glucose analog [^18^F]fluorodeoxyglucose (^18^F-FDG) can be used to diagnose [1], grade [2], stage [3, 4], and to assess treatment response [5–7] in soft tissue and bone sarcomas. We have recently reported that treatment monitoring with FDG-PET in patients with soft tissue sarcoma provides accurate response information (as determined by %-necrosis in excised tumors) after the initial cycle [5] and after completion [8] of neoadjuvant therapy when thresholds of 35% and 60% reductions in tumor FDG SUV were applied.

 Histopathologic response to neoadjuvant therapy is a well established and significant prognostic factor for disease-free and overall survival in patients with bone sarcomas [9, 10]. Bone sarcomas tend to have higher response rates to neoadjuvant treatment than soft tissue sarcomas with 5-year disease-free survival rates of 60%–75% [11]. In agreement with previous observations in patients with soft tissue sarcomas [8], a study in bone sarcomas has suggested that a reduction of tumor metabolic activity by more than 60% at the end of treatment accurately predicts histopathologic tumor response [7]. For the current study we tested whether applying this threshold of 60% reduction in tumor FDG SUVmax after completion of therapy in a prospectively enrolled study group yielded reliable histopathologic response predictions in patients with bone sarcomas.

## 2. Material and Methods

### 2.1. Patients and Study Design

From February 2005 to November 2007, twelve adult patients with resectable high-grade bone sarcomas were prospectively enrolled in this study. Participants underwent a pretreatment (baseline) PET/CT scan and a followup scan after the completion of neoadjuvant therapy. The study population consisted of 7 females and 5 males with a mean age of 31.6 ± 15.0 years (range, 18–61 years). All patients presented with primary, nonmetastatic disease at the time of the first PET/CT scan. 

The study group consisted of six patients with osteosarcoma (50%), 3 with Ewing's sarcoma (25%), 2 with dedifferentiated chondrosarcoma (17%), and 1 with a malignant giant cell tumor of the bone (8%). 

**Figure 1:**
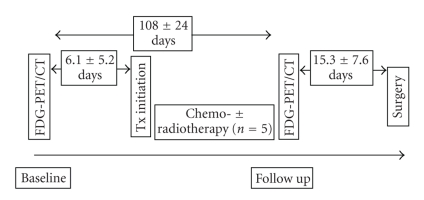
Study design.

A baseline FDG-PET/CT scan was performed prior to the initiation of neoadjuvant therapy after a mean interval of 6.1 ± 5.2 days (range, 1–15). A followup scan after completion of neoadjuvant treatment was performed in all patients after a mean interval of 15.3 ± 7.6 days (range, 3–24 days) prior to surgical resection. The study design is depicted in Figure 1.

All participants gave written informed consent after the details of the study were explained by a study physician. The study was approved by the UCLA Institutional Review Board.

### 2.2. Neoadjuvant Therapy

The neoadjuvant treatments varied with the bone sarcoma subtype. Patients with Ewing's sarcoma were treated with alternating cycles of VAC (vincristine, adriamycin, cytoxan) and IE (ifosfamide, etoposide) [11]. Patients underwent 4 cycles (2 VAC and 2 IE) prior to surgical resection. Patients with osteosarcoma underwent treatment protocols that utilized a 3-drug regimen in the neoadjuvant setting. This consisted of two cycles of chemotherapy. Each cycle consisted of doxorubicin (75 mg/m^2^) and cisplatin (100 mg/m^2^) followed by two doses of high-dose methotrexate (12 grams/m^2^) with leucovorin rescue. If age or performance status prevented the use of methotrexate, two cycles of doxorubicin and cisplatin were given prior to resection. Patients with the malignant giant cell tumor and the dedifferentiated chondrosarcomas received ifosfamide and doxorubicin-based chemotherapy. Five of 12 patients (42%) received additional neoadjuvant external beam radiation.

### 2.3. PET/CT Image Acquisition and Reconstruction

PET/CT studies were performed using a hybrid device consisting of a PET scanner with LSO detectors (ECAT ACCEL) and a dual detector helical CT scanner (Siemens Biograph Duo PET/CT scanner). 

To standardize imaging conditions and to assure that elevated blood glucose levels would not affect FDG uptake measurements, patients were instructed to fast for at least 6 hours prior to FDG-PET/CT imaging. Blood glucose was measured before the injection of FDG (mean: 96 ± 16 mg/dL; range: 78–141 mg/dL). 

The CT acquisition parameters were: 130 kVp, 120 mAs, 1 second rotation, and 4 mm slice collimation, 8-mm/s bed speed. The CT portion of the study was performed with the administration of intra-venous contrast (Omnipaque, Novaplus) in 11 patients at baseline and 8 patients at followup. In addition, 12 patients received oral contrast at baseline and 11 patients at followup, respectively.

Approximately sixty minutes before image acquisition patients were injected intravenously with 0.21 mCi/kg of ^18^F-FDG. Depending on the patient's body weight PET emission scan duration per bed position varied between 1 to 5 minutes as previously described [12, 13]. Care was taken to assure a similar time interval from injection of FDG to the start of imaging for baseline and followup studies.

The CT images were reconstructed using conventional filtered back-projection, at 3.4 mm axial intervals to match the slice separation of the PET data. 

PET images were reconstructed using iterative algorithms (OSEM two iterations, eight subsets). To correct for photon attenuation a previously published CT-based algorithm was applied [14].

### 2.4. Image Analysis

The Mirada workstation (REVEALMVS; CTI Mirada Solutions) was used to view PET and CT images and to measure tumor FDG uptake. All FDG images were analyzed by one observer who was blinded to histopathologic treatment response. The single maximum pixel value within the slice with the highest radioactivity concentration was detected on baseline and followup scans as previously described [15]. The maximum standardized uptake value (SUVmax) was calculated by dividing the activity in that pixel by the administered dose normalized to patient body weight (g/mL). One radiologist blinded to PET and histopathological response data measured tumor diameters on baseline and followup CT images.

### 2.5. Metabolic Response Criteria

As previously reported, a reduction in tumor SUV by ≥60% after the course of neoadjuvant treatment accurately predicts histopathologic tumor response in sarcomas [7, 8]. We therefore applied this threshold prospectively in the current study population.

### 2.6. Reference Standard

Following excision, a complete cross section of tumor was submitted in multiple cassettes for histopathologic evaluation. The fraction of tissue with treatment effect (necrosis and/or fibrosis) of the total tumor tissue served as reference standard. This is based on previous studies demonstrating that >90% treatment effect in excised tissue after neoadjuvant treatment is associated with favorable survival in bone sarcoma patients [9, 10]. 

### 2.7. Histopathology

Each sarcoma subtype showed characteristic histopathologic features, as described in standard texts. Pathologic diagnoses were made by a pathologist (S.D.) with expertise in sarcoma pathology who was blinded to PET data. Osteosarcomas consisted of malignant osteoblasts with associated malignant osteoid formation. Ewing's sarcomas showed sheets of small round blue cells with scant cytoplasm and strong CD99 membrane positivity. Dedifferentiated chondrosarcomas showed a biphasic pattern with foci of hyaline cartilage, with scattered atypical chondrocytes, admixed with a nonchondroid high-grade sarcoma. The malignant giant cell tumor showed a mixture of giant cells with pleomorphic nuclei and atypical mitoses and admixed ovoid stromal cells also with atypical nuclei and mitoses; an extensive panel of immunohistochemical stains in this case was all negative. A complete cross section of each resected tumor was submitted in multiple cassettes. Treatment effect, indicated by tumor necrosis and fibrosis, was evaluated in each case.

### 2.8. Statistical Analysis

Quantitative data are presented as mean ± SD, median, and range. The Mann-Whitney test and the Wilcoxon signed rank test were used for unpaired and paired comparisons between quantitative parameters. The Pearson correlation was performed to correlate changes in SUVmax with percent of histopathologic necrosis in the excised tumor. *P* values <.05 were considered statistically significant except for the bivariate correlation (correlation is significant at the 0.01 level). Statistical analyses were performed using SPSS software for Windows (version 14.0, SPSS Inc., Chicago, USA) and Statistica software for Windows (version V8.0StatSoft, Inc., Tulsa, USA).

## 3. Results

### 3.1. Changes in Tumor Diameter

Baseline and followup CT tumor size measurements are listed in Table 1. 

Mean tumor size at baseline averaged 11.7 ± 5.1 cm (median, 10.1 cm; range, 6.5–22.1 cm) and 11.3 ± 4.9 cm (median, 9.5 cm; range, 6.5–21.8 cm) at followup. Tumor size did not change significantly during the course of treatment (*P* = .37).

### 3.2. Histopathologic Response

The necrotic fraction of the tumors averaged 61 ± 31% of the entire tumor volume (median, 70%, range, 10%–99%). Four of 12 patients (2 Ewing's sarcomas, 2 Osteosarcomas) exhibited ≥90% treatment effect in the resected specimen and were therefore classified as histopathologic responders (33% response rate). Five patients (42%) exhibited ≤50%, and 3 patients (25%) between 50 and 90% treatment effect after neoadjuvant therapy. These patients were classified as histopathologic nonresponders.

### 3.3. Changes in Tumor Size and Histopathologic Response

Tumor size changes in response to treatment were small and did not differ significantly among histopathologic responders (*n* = 4) and nonresponders (*n* = 8)  (−1 ± 4% versus 4 ± 9%; *P* = .16) (Figure 3).

### 3.4. Changes in Tumor FDG Uptake

Baseline and followup SUVmax measurements are listed in Table 1. SUVmax decreased by 40 ± 31% from 8.1 ± 4.1 g/mL at baseline to 4.5 ± 2.7 g/mL at late followup (*P* = .006). Tumor SUVmax and changes in SUVmax did not differ significantly among the sarcoma subtypes (*P* = NS).

### 3.5. Changes in FDG Uptake and Histopathologic Response

**Figure 2:**
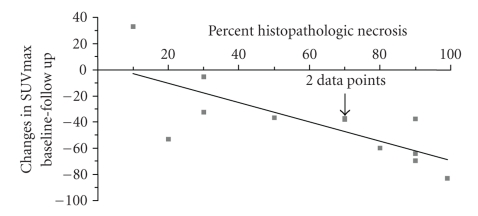
Changes in SUVmax from baseline to late followup are significantly correlated with percent of histopathologic necrosis in the excised tumor tissue (Pearson Correlation Coefficient = −0.75; *P* = .005).

Histopathologic responders showed significantly more pronounced decreases in tumor FDG uptake from baseline to late followup compared to nonresponders (64 ± 19% versus 29 ± 30%; *P* = .03). Changes in SUVmax in response to treatment were significantly correlated with percent necrosis in the excised tumor (Pearson Correlation Coefficient = −0.75; *P* = .005; Figure 2).

A 60% decrease in SUVmax from baseline to late followup as threshold criterion correctly classified 3 of 4 histopathologic responders as metabolic responders (sensitivity, 75%) and 7 of 8 histopathologic nonresponders as metabolic nonresponders (specificity, 88%).

**Figure 3:**
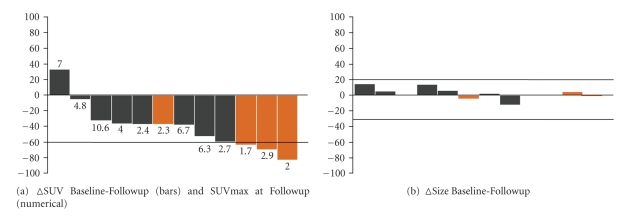
(a) SUVmax values (numerical) and changes in SUVmax (bars) after completion of neoadjuvant treatment for each patient. (b) depicts changes in tumor size from baseline to end of treatment. Histopathologic responders are illustrated in orange. Three of four histopathologic responders showed decreases in SUVmax by ≥60% from baseline to followup scan. One histopathologic responder with a 38% decrease in FDG uptake from baseline to followup showed an SUVmax value after completion of neoadjuvant therapy of <2.5 and was therefore correctly classified. Size changes in response to treatment were marginal.

A single histopathologic responder and one histopathologic nonresponder were misclassified as metabolic nonresponder and responder, respectively (Figure 3; Table 2).

## 4. Discussion

The current prospective study demonstrates that post-neoadjuvant treatment FDG-PET evaluations in adult patients with bone sarcomas identified histopathological treatment responders accurately when a prospective cut-off of ≥60% reductions in SUVmax was applied. 

The inability of changes in tumor size to distinguish treatment responders from nonresponders is not surprising and has been well established in bone sarcomas and various types of cancers [16]. Parameters assessed by conventional radiography such as tumor location, tumor size, radiographic appearance, margination, cortical destruction, and periosteal reaction are of limited use in assessing therapeutic responses [17].

As a prerequisite for monitoring of treatment effects most bone sarcomas exhibit markedly increased FDG-uptake with a mean baseline SUVmax of 8.1 ± 4.1 g/mL (range, 3.6 to 15.7 g/mL). This observation is in concordance with two previsously published papers that describe mean SUVs of 9.6 and 7.9, respectively [3, 18]. Thus, it should be feasible to determine changes in FDG uptake in response to treatment and to use this information for predicting treatment responses. This has been reported in some earlier prospective and retrospective studies [7, 18–22]. Schulte et al. [7] observed in 27 patients with osteosarcoma that a decrease in FDG SUV tumor to background ratio from baseline to end of treatment by at least 60% detected histopathologic response with a sensitivity of 100% and a specificity of 80%. 

In the current study we prospectively applied this previously reported threshold as a response criterion. A 60% threshold for a metabolic response correctly classified 7 of 8 histopathologic nonresponders as metabolic nonresponder (specificity, 88%) and 3 of 4 histopathological responders as metabolic responders (sensitivity, 75%). The single “false negative” response by PET occurred in the patient with the lowest baseline tumor FDG uptake of all study participants (SUVmax at baseline: 3.6 g/mL). In this patient SUVmax decreased from baseline to late followup by only 38%, to 2.3 g/mL. This false negative result is consistent with the notion that response assessments by FDG-PET are less reliable in tumors with lower FDG baseline uptake [23]. 

In a subgroup analysis that excluded patients with chondrosarcoma and giant cell tumor the sensitivity of FDG-PET in assessing histopathologic response was identical (sensitivity, 75%). However, excluding patients with those subtypes resulted in a decreased specificity (80% versus 88% resp.).

In a retrospective analysis of 36 patients with Ewing sarcoma family of tumors, Hawkins et al. [18] found that a decrease in SUV ≥50% and an end of treatment SUVmax of <2.5 g/mL detected histopathologic response with a sensitivity of 83% and 76%, respectively. Despite the superiority of changes in SUV in the detection of histopathologic responses, an end of treatment SUV of less than 2.5 g/mL was more predictive of progression free survival in their study. We therefore tested whether an end of treatment tumor SUVmax of <2.5 could also identify treatment responders. This resulted in a sensitivity of 75% and a specificity of 88% for predicting tissue responses. However, the combination of both response criteria (response: ≥60% decrease in SUVmax or SUVmax at late followup <2.5; nonresponse: <60% decreases in SUVmax and SUVmax at late followup >2.5) correctly identified all histopathological responder as metabolic responder (sensitivity, 100%) and 6 of 8 histopathologic nonresponder as metabolic nonresponder (specificity, 75%).

**Figure 4:**
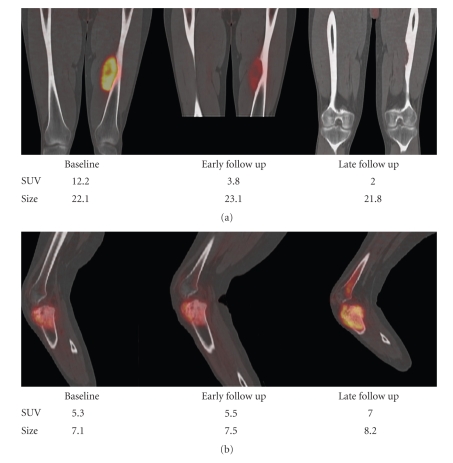
FDG-PET/CT at baseline, early followup, and after completion of neoadjuvant treatments in a histopathological responder (a) and a nonresponder (b).

End of treatment evaluations has limited impact on patient management. We have previously shown that reductions in SUVmax by ≥35% after the initial cycle of neoadjuvant treatment predicts histopathological responses in soft tissue [5] sarcomas. In another prospective study at our institution such early response assessments were obtained in 4 patients with bone sarcomas. A metabolic response of 35% correctly classified 1 of 1 histopathologic responder as metabolic responder and 2 of 3 histopathologic nonresponders as metabolic nonresponders (Figure 4). Future studies need to determine whether such early response predictions hold up in larger patient populations. 

PET data can be analyzed semiquantitatively. This allows for a reliable definition of metabolic tumor activity before, during, and after neoadjuvant therapy. However, few studies have attempted to apply response criteria across institutions and study populations. The current study adds to a growing body of evidence established in sarcoma and other cancers that late reductions in SUV by ≥60% can accurately predict responses to neoadjuvant therapy [7, 8, 24, 25].

Besides the evaluation of response/nonresponse by PET, new imaging modalities such as dynamic magnet resonance imaging [26] and new image analysis methods/response criteria [27] are currently under investigation for early response assessments in sarcoma patients. Previously published data suggest that a combination of changes in tumor density (>15% change) and in modified tumor size (>10%) accurately detects early response in gastrointestinal stromal tumors (GIST) to Imatinib treatment [27]. Whether these imaging modalities or response criteria are superior over PET and the proposed definition of a metabolic response needs to be addressed in future studies.

The primary limitation of this study is the small study population. However, we felt that reporting this trial adds to growing body of literature that addresses the ability of FDG-PET/(CT) for monitoring treatment responses in sarcoma patients. Larger prospective studies are needed to confirm these data. Another limitation was the heterogeneity of the study population with regards to tumor histology and treatment regimens. However, we believe that any useful imaging test used for monitoring therapeutic interventions needs to be applied across disease subtypes and treatment approaches. 

In conclusion, serial FDG-PET imaging accurately classified histopathologic response/nonresponse to neoadjuvant therapy in adult primary bone sarcomas. It is noteworthy that a combination of previously applied response criteria (reductions in SUVmax by ≥60% and a posttreatment tumor SUVmax of <2.5) provided the best response predictions. This information could now be applied prospectively to examine whether PET-guided therapeutic decisions affect survival of bone sarcoma patients.

## Figures and Tables

**Table 1:** Patient characteristics and imaging findings.

*n*	Subtypes	Location	Age	Sex	Baseline		End of treatment		Metabolic response (decrease ≥60%)	Necrosis
					SUVmax	Size	SUVmax	Size		
1	Ewing's Sarcoma*	Femur	22	M	12.2	22.1	2.0	21.8	Yes	99
2	Ewing's Sarcoma*	Fibula	19	F	4.7	7.8	1.7	7.8	Yes	90
3	Osteosarcoma	Fibula	23	M	3.6	6.8	2.3	6.5	No	90
4	Osteosarcoma	Tibia	58	M	9.7	8.5	2.9	8.8	Yes	90
5	Ewing's Sarcoma*	Femur	29	M	6.8	17.3	2.7	17.3	Yes	80
6	Osteosarcoma	Femur	31	F	10.8	15.0	6.7	15.3	No	70
7	Osteosarcoma	Tibia	18	F	3.9	11.2	2.4	11.9	No	70
8	Giant Cell Tumor*	Iliac Wing	44	F	6.4	9.3	4.0	10.5	No	50
9	Osteosarcoma	Femur	32	F	5.0	6.5	4.8	6.8	No	30
10	Chondrosarcoma*	Humerus	61	M	15.7	17.4	10.6	#	No	30
11	Chondrosarcoma	Sternum	19	F	13.5	10.9	6.3	9.5	No	20
12	Osteosarcoma	Tibia	23	F	5.3	7.1	7.0	8.2	No	10

*Patients received additional radiation therapy; # not measurable due to obliquity of bone.

**Table 2:** Correlation between histopathologic and metabolic response (≥60% decreases in SUVmax).

	Histopathology		
Metabolic response	Responder (≥90% necrosis)	Nonresponder (<90% necrosis)	
Responder (≥60% decrease in SUVmax)	3	1	Positive predictive value 3/4 = 75%
Nonresponder (<60% decrease in SUVmax)	1	7	Negative predictive value 7/8 = 88%
	Sensitivity 3/4 = 75%	Specificity 7/8 = 88%	Accuracy 10/12 = 83%
